# Non-Coding RNAs and Lipid Metabolism

**DOI:** 10.3390/ijms150813494

**Published:** 2014-08-04

**Authors:** Elisabeth Smolle, Johannes Haybaeck

**Affiliations:** Institute of Pathology, Medical University Graz, Auenbruggerplatz 25, A-8036 Graz, Austria; E-Mail: elisabeth.smolle@stud.medunigraz.at

**Keywords:** non-coding RNAs, micro RNAs, long non-coding RNAs, dyslipidaemia

## Abstract

A high percentage of the mammalian genome consists of non-coding RNAs (ncRNAs). Among ncRNAs two main subgroups have been identified: long ncRNAs (lncRNAs) and micro RNAs (miRNAs). ncRNAs have been demonstrated to play a role in a vast variety of diseases, since they regulate gene transcription and are involved in post-transcriptional regulation. They have the potential to function as molecular signals or as guides for transcription factors and to regulate epigenetic modifiers. In this literature review we have summarized data on miRNAs and lncRNAs and their involvement in dyslipidaemia, atherosclerosis, insulin resistance and adipogenesis. Outlining certain ncRNAs as disease biomarkers and/or therapeutic targets, and testing them *in vivo*, will be the next steps in future research.

## 1. Introduction

A large percentage of the mammalian genome consists of non-coding RNAs (ncRNAs) [1,2,3].

Among ncRNAs there are two main subgroups: long ncRNAs (lncRNAs) and micro RNAs (miRNAs). ncRNAs appear in many forms: they may be very short, or a few hundred kilobases in length. Some of them are spliced, whilst others are unspliced. ncRNAs can form linear or tertiary structures, and some of them interact with DNA, proteins or RNA [4].

lncRNAs were initially discovered in the 1990s, when it was found that the lncRNA XIST (“X-chromosome inactivation”) inactivates the X-chromosome in females [5,6]. lncRNAs measure between 200 nucleotides and >100 kilobases, and they are commonly composed of one gene structure comprising up to 14 exons [1,7]. lncRNAs are produced either within their target gene or in the vicinity of the target gene; this is a key feature of lncRNAs [8]. They are divided into five groups, corresponding to their association with mRNA: (1) sense; (2) antisense (the coding transcript and the lncRNA-transcript are reversed); (3) intronic (lncRNAs that stem from the introns of another transcript); (4) intergenic (lncRNAs located between two genes); (5) bidirectional (lncRNAs in the reverse direction with one or more exons in the same chain) [9].

The number of lncRNAs in the genome is still debated, but it is estimated that there are >60,000 lncRNAs in the human genome [10,11]. Similarities between lncRNAs and protein-coding transcripts have been reported, e.g., lncRNAs are transcribed by RNA-polymerase [12], they are spliced at canonical splicing sites [13], are partly polyadenylated [14] and associated with polysomes [15]. By contrast, certain characteristics of lncRNAs differ from protein-coding genes: lncRNAs are expressed at lower levels, are less evolutionary conserved and less frequently associated with ribosomes compared to protein-coding transcripts [10]. lncRNAs are known to regulate gene transcription and to be involved in post-transcriptional regulation. They have the potential to function as molecular signals or guides for transcription factors and to regulate epigenetic modifiers [2]. lncRNAs themselves encode miRNAs and target certain mRNAs leading to RNA decay [2]. It is suggested that lncRNAs play a role in gene expression changes in response to extracellular stimuli [16,17,18,19]. According to Wang *et al.* [1], lncRNAs promote the timing of gene expression. Notably, some lncRNAs are able to generate small peptides and are thus acting as both, coding and non-coding, transcripts [7].

miRNAs are short single-stranded ncRNAs, measuring between 19 and 25 nucleotides in length, and are well preserved in the eukaryotic genome [20,21]. miRNAs form base pairs with complementary loci within target mRNAs, and thereby negatively regulate gene expression via inhibition of translation and induction of specific mRNA degradation [20,22,23]. Although miRNAs were discovered in the 1990s, they were not recognized as post-transcriptional regulators until the 2000s [24,25,26,27]. miRNAs are potent endogenous regulators of gene expression, as every given miRNA has multiple gene targets [28]. A single miRNA has the potential to regulate an entire cellular pathway, and any miRNA may contain multiple mRNA binding sites, which also contribute to miRNA-mediated gene silencing [28]. Specific subsets of miRNAs are aberrantly expressed, dependent on the biological conditions in different tissues, meaning that regulation of gene expression by miRNAs is context-dependent [28].

miRNAs are encoded by genes [27] and either transcribed from their own promoters or within a host protein-encoding gene [20,24,29], mediated by RNA polymerase II, which leads to the formation of a primary miRNA transcript (pri-miRNA) [24,30]. Double-stranded pri-miRNA is then cleaved by a nuclear ribonuclease complex, generating a precursor miRNA (pre-miRNA) [30,31]. After that, the pre-miRNA is exported to the cytoplasm, where the pre-miRNA undergoes further enzymatic processing, and the final short double-stranded miRNA strand is generated [32,33,34]. The miRNA seed region (nucleotides at position 2–8) is involved in gene silencing, interacting with the target mRNA, which predominantly leads to a down-regulation of this target mRNA [35,36]. Circulating extracellular miRNAs, occurring stably in blood plasma without an association with cells, have been discovered [37]. The levels of these extracellular miRNAs are associated with disease states [37,38,39]. For example, extracellular miRNAs have been demonstrated to be associated with lipid-based carriers, such as high-density lipoproteins (HDLs) [40,41]. HDLs contain miRNA signatures and miRNA profiles of HDL are altered in humans and mice suffering from cardiovascular disease [41].

## 2. In Depth Review of Existing Data

### 2.1. Dyslipidaemia

miRNAs are associated with plasma HDL cholesterol, and HDL cholesterol levels are known to inversely correlate with cardiovascular diseases [27,42]. HDL-association of miRNAs and the role of HDLs in miRNA transport link miRNAs to cardiovascular disease [27]. Cholesterol biosynthesis in the liver is important for the synthesis of lipids and lipoproteins in general, and for a proper function of lipid metabolism [43,44]. Recently, it has been demonstrated that miRNAs modulate these processes [45,46,47]. The *miR-27b* gene has been identified as a central candidate gene, regulating lipid metabolism post-translationally [47]. *miR-27b* was determined to target several lipid-associated genes in human hepatoma cell lines, for example heparan sulfate *N*-deacetylase/*N*-sulfotransferase 1 (*NDST1*), angiopoietin-like 3 (*ANGPTL3*), peroxisome proliferator-activated receptor γ (*PPARG*) and glycerol-3-phosphate acyltransferase 1 (*GPAM*). Vickers and colleagues demonstrated that *miR-27b* and its target genes were significantly modulated in murine liver tissue, responding to diet-induced hyperlipidaemia [47]. As a reaction to elevated plasma triglycerides (TGs) and liver steatosis, hepatic *miR-27b* was significantly increased, and the ANGPTL3 and GPAM protein, being targets of *miR-27b*, were significantly repressed [47]. As a reaction to hypertriglyceridaemia and hepatic steatosis, hepatic *miR-27b* increases, which leads to an inhibition of *de novo* biosynthesis of TGs [47].

The PPARα protein mainly occurs in the liver and is a regulator of multiple genes involved in fatty acid transport, catabolic processes and energy supply [48]. *miR-21* and *miR-27b* both negatively regulate PPARα [48], and PPARα protein levels in human liver-derived cell lines are decreased by over-expression of *miR-21* and *miR-27b* [48]. Zheng *et al.* [49] found PPARα to be regulated by *miR-10b* in a human hepatocyte cell line. In this study, human hepatocyte L02 cells were cultured with an abundance of non-esterified fatty acids, as a non-alcoholic fatty liver disease (NAFLD) model. Upon treatment with these non-esterified fatty acids, *miR-10b* levels were up-regulated in hepatocytes [49]. The PPARα (*PPARA*) gene is a target of *miR-10b*, and thus, PPARα protein levels are significantly decreased in the L02 cells. It is probable that *miR-10b* and its regulation of PPARα is a condition occurring in certain liver diseases, such as NAFLD [49].

miRNAs also play a considerable role in cholesterol metabolism [27]. First of all, *miR-33a* and *miR-33b* have been investigated with regards to cholesterol [50,51,52,53,54,55]. *miR-33a* is located within intron 16 of sterol-regulatory-element-binding protein 2 (*SREBP2*), which is a central transcription factor in lipid metabolism [51,52,55]. When cholesterol is low, SREBP2 is released from the endoplasmic reticulum and enters the nucleus where it activates and increases cellular cholesterol synthesis, leading to increased low-density lipoprotein (LDL) cholesterol uptake [56]. SREBP2 and *miR-33a* are activated consecutively, and *miR-33a* directly targets and diminishes ATP-binding cassette transporter A1 (*ABCA1*) mRNA levels, thus repressing cholesterol efflux from the cells. Since *miR-33a* and *miR-33b* are located in the introns of the *SREBP* genes, they are involved in controlling cholesterol and lipid metabolism. *miR-33a/b* also targets ATP-binding cassette transporter G1; cholesterol efflux (*ABCG1*), Niemann-Pick C1; cholesterol storage (*NPC1*), ATP-binding cassette transporter B11; bile secretion (*ABCB11*) and phospholipid transporting ATPase IC; bile acid secretion (*ATP8B1*) [51,52,55,57]. These genes are all involved in cholesterol metabolism. Furthermore, it was found that *miR-33a/b* play a central role in fatty acid β-oxidation, because they target carnitine *O*-octanyl transferase (*CROT*), carnitine palmitoyltransferase 1A (*CPT1A*) and hydroxyacyl-CoA dehydrogenase-3-ketoacyl-CoA thiolase-enoyl-CoA hydratase β-subunit (*HADHB*) [53,58]. In a study on non-human primates, the inhibition of *miR-33a/b* was associated with reduced VLDL (very low-density lipoprotein) secretion and reduced plasma TG levels [59]. *miR-122* was also found to influence lipid metabolism, since anti-*miR-122* therapy reduced cholesterol levels in mice and primates significantly [45,60,61]. Most likely, *miR-122* down-regulates genes playing a key-role in cholesterol metabolism, such as 3-hydroxy-3-methylglutaryl-CoA reductase (*HMGCR*) and 3-hydroxy-3-methylglutaryl-CoA synthase 1 (*HMGCS1*) [62,63,64]. *miR-122-*deficient mice had a significantly reduced serum TG rate, suggesting that *miR-122* is involved in TG metabolism [64].

Recently it was found that *miR-144* regulates the expression of *ABCA1* in macrophages and hepatocytes, and over-expression of *miR-144* in murine macrophages inhibits ABCA1 protein expression [65]. It was demonstrated that over-expression of *miR-144* reduces, and inhibition of *miR-144* increases, circulating HDL levels in mice [65]. Other miRNAs that play essential roles in lipid metabolism by targeting and repressing *ABCA1* in various cell types are *miR-106*, *miR-26* and *miR-758* [66,67,68]. *miR-1*, *miR-206* and *miR-613* have been reported to repress lipogenesis by targeting liver X receptor alpha (LXRα) [69,70]. *miR-146α* has been shown to repress Toll-like receptor 4 (TLR4) signaling, and to inhibit oxidized LDL (oxLDL) cholesterol uptake in macrophages [71].

*miR-155*, a miRNA that is usually associated with inflammation, has been reported to repress lipid uptake in oxLDL-stimulated dendritic cells, because it targets and down-regulates the scavenger receptor, CD36, and the lectin-type oxidized LDL receptor 1 (LOX-1). oxLDL stimulation was found to up-regulate *miR-155*, together with *miR-9*, *miR-146a*, *miR-125a-5p* and *miR-146-5p*, in monocytes [72]. Chen *et al.* [72] found, that *miR-125-5p* was repressing lipid uptake by targeting oxysterol-binding protein-like 9 (ORP9). In another study it was shown that *miR-155* increased both lipid uptake and inflammation in oxLDL-stimulated macrophages [73]. *miR-125*, *miR-455-5p*, *miR-185*, *miR-96* and *miR-233* have been reported to reduce HDL cholesterol uptake [41,74,75]. In a recent study, *miR-217* was found to influence the repression of ethanol-induced sirtuin 1 (*SIRT1*) and to promote ethanol-induced lipid accumulation and fatty acid synthesis [76]. According to this study, *miR-217* plays a central role in fatty acid oxidation and in fatty acid synthesis [76].

Single nucleotide polymorphisms have recently been identified within the miRNAs and in the miRNA target sites [77,78,79,80,81]. For example, a gain-of-function variant in the lipoprotein lipase (*LPL*) gene apparently abolishes a *miR-410* target site [82].

Summarizing these findings, it is obvious that a vast variety of miRNAs play a role in dyslipidaemia and related processes. Whether any of these miRNAs can be used as disease biomarkers or therapeutic targets remains to be investigated in detail. Table 1 summarizes the most notable data concerning miRNAs and their role in dyslipidaemia (Table 1).

**Table 1:** Micro RNAs and their involvement in dyslipidaemia.

miRNA	Target	Site	Effect	References	
*miR-27b*	Heparan sulfate *N*-deacetylase/*N*-sulfotransferase 1 (NDST1), angiopoietin-like 3 (ANGPTL3), peroxisome proliferator-activated receptor γ (PPARG), glycerol-3-phosphate acyltransferase 1 (GPAM)	Murine liver tissue	As reaction to elevated plasma triglycerides (TGs) and liver steatosis, hepatic *miR-27b* was increased, and the ANGPTL3 and GPAM protein were repressed. As reaction to hypertriglyceridaemia and hepatic steatosis, hepatic *miR-27b* increases, leading to an inhibition of de novo biosynthesis of TGs.	Vickers 2013 [47]	
*miR-21*, *miR-27b*	PPARα protein	Human liver-derived cell lines	PPARα protein levels in human liver-derived cell lines were decreased by over-expression of *miR-21* and *miR-27b*.	Kida 2011 [48]	
*miR-10b*	PPARα protein	Human hepatocyte cell line	Human hepatocyte L02 cells were cultured with an abundance of non-esterified fatty acids, as a non-alcoholic fatty liver disease (NAFLD) model. Upon this treatment *miR-10b* levels were up-regulated. PPARα (PPARA) was decreased at protein level in the L02 cells.	Zheng 2010 [49]	
*miR-33a*, *miR-33b*	ATP-binding cassette transporter G1; cholesterol efflux (ABCG1), Niemann-Pick C1; cholesterol storage (NPC1), ATP-binding cassette transporter B11; bile secretion (ABCB11), phospholipid transporting ATPase IC; bile acid secretion (ATP8B1)	Liver (human)	Regulation of cholesterol homeostasis, regulation of cholesterol transport. Expression of *miR-33* inhibits cholesterol export and fatty acid oxidation.	Rayner 2010 [51], Marquart 2010 [52], Najafi-Shoushtari 2010 [55], Allen 2012 [57]	
*miR-33a*, *miR-33b*	Carnitine *O*-octanyl transferase (CROT), carnitine palmitoyltransferase 1A (CPT1A), hydroxyacyl-CoA dehydrogenase-3-ketoacyl-CoA thiolase-enoyl-CoA hydratase β-subunit (HADHB)	Liver (non-human primates)	Regulation of fatty acid β-oxidation. Inhibition of *miR-33a/b* was associated with reduced VLDL (very-low-density lipoprotein) secretion and reduced plasma TG levels.	Gerin 2010 [53], Davalos 2011 [58]	
*miR-122*	3-hydroxy-3-methylglutaryl-CoA reductase (HMGCR), 3-hydroxy-3-methylglutaryl-CoA synthase 1 (HMGCS1)	Liver (mice, primates)	Anti-miR-122 therapy reduced cholesterol levels in mice and primates. miR-122 down-regulates genes playing a key-role in cholesterol metabolism such as HMGCR and HMGCS1 miR-122-deficient mice had a significantly reduced serum TG rate.	Esau 2006 [45], Elmen 2008 [60,61], Krutzfeld 2005 [62], Hsa 2012 [63], Tsai 2012 [64]	
*miR-144*	ATP-binding cassette transporter A1 (ABCA1)	Liver (mice)	miR-144 regulates the expression of ABCA1 in macrophages and hepatocytes. Over-expression of miR-144 in murine macrophages inhibits ABCA1 protein expression. Over-expression of miR-144 reduces, and inhibition of miR-144 increases circulating HDL levels in mice.	Ramirez 2013 [65]	
*miR-1*, *miR-206*, *miR-613*	Liver X receptor alpha (LXRα)	Human hepatocytes	Repress lipogenesis by targeting liver X receptor alpha (LXRα).	Zhong 2013 [69,70]	
*miR-146α*	Toll-like receptor 4 (TLR4)	Human macrophages	miR-146α represses Toll-like receptor 4 (TLR4) signaling, and inhibits oxidized LDL (oxLDL) cholesterol uptake in macrophages.	Yang 2011 [71]	
*miR-155*	Scavenger receptor, CD36; lectintype oxidized LDL receptor 1 (LOX-1)	Human dendritic cells	miR-155 represses lipid uptake in oxLDL-stimulated dendritic cells.	Chen 2009 [72]	
*miR-125-5p*	Oxysterol-binding protein-like 9 (ORP6)	Human macrophages	miR-125-5p represses lipid uptake in macrophages.	Chen 2009 [72]	

### 2.2. HDL (High-Density Lipoprotein) Cholesterol and Extracellular miRNAs

Extracellular miRNAs are detectable in the blood plasma and are protected from degradation, because of packaging in microvesicles, exosomes and apoptotic bodies, or because of their linkage to proteins [83]. HDL particles were found to transport endogenous miRNAs, and deliver them to recipient cells [41]. In individuals suffering from familial hypercholesterolaemia (FH), HDL-miRNA profiles significantly differ from healthy controls, indicating that the function of miRNAs is altered when carried by HDL [41]. FH-HDL was found to have a greater concentration of miRNAs that are most abundantly found in normal HDL, and FH-HDL also contained more individual miRNAs compared to normal HDL. In a study where normal HDL was enriched with the most abundant FH-HDL miRNA, *hsa-miR-233*, it was shown that the recipient cells had increased intracellular *miR-223* levels and that *miR-233* target genes were reduced, such as member B of the Ras homolog gene family (*RhoB*), and Ephrin A1 (*EFNA1*). When FH-HDL-miRNAs were introduced to hepatocytes in cell culture, a significant loss of conserved mRNA targets compared with normal HDL-miRNAs was observed. The down-regulated genes were mostly (79 out of 91) potential targets of miRNAs that were abundantly present in FH-HDL [41]. It was recently discovered that the properties of HDL cholesterol, e.g., anti-oxidative, anti-inflammatory and anti-thrombotic, can be very heterogeneous and that HDL cholesterol function may be altered in individuals suffering from cardiovascular disease or diabetes [84]. Thus, it is possible that HDL-carried miRNAs contribute to this heterogeneity of HDL function, altering the effect of HDL on endothelial cells and macrophages.

### 2.3. Vascular Inflammation

For atherogenesis, vascular inflammation is the first event to take place, leading to the development of an atherosclerotic lesion. In this process, proinflammatory cytokines, like tumor necrosis factor-alpha (TNF-α), enhance the expression of adhesion molecules in endothelial cells and thus lead to the recruiting of inflammatory cells to the inflammation site [85,86]. The TNF-α-mediated activation of endothelial cells has been demonstrated to decrease *miR-181b* expression [68]. It was evident that both *in vitro* and *in vivo* over-expression of *miR-181b* blocks the expression of adhesion molecules, like vascular adhesion molecule 1 (VCAM-1). Interestingly, *miR-181b* leads to a decrease in endothelial cell activation and leukocyte recruiting in lipopolysaccharide-induced lung injury, when administered systemically. Furthermore, *miR-181b* has been shown to diminish the nuclear translocation of nuclear factor kappa B (NF-κB), targeting importin-α3. It is therefore likely that the inhibitory effect of *miR-181b* on the expression of adhesion molecules results from the inhibition of NF-κB nuclear translocation [68].

According to Harris *et al.* [87], *miR-126* and *miR-195* are also involved in vascular inflammation. In fact, *miR-126* is abundantly expressed in endothelial cells and oppresses their VCAM-1 expression. *miR-126* also decreases leukocyte binding to TNF-α-activated endothelial cells [87].

In a study by Hu *et al.* [88], it was demonstrated that down-regulation of *miR-144-3p*, a miRNA targeting *ABCA1* which mediates reverse cholesterol transport, resulted in plaque formation. In this study, *ABCA1* was identified as a potential target of *miR-144-3p*, since *ABCA1* was down-regulated after transfection of cells with *miR-144-3p* mimics. The *miR-144-3p* mimics enhanced the expression of inflammatory factors, namely interleukin 1β, interleukin 6 and TNF-α, and blocked cholesterol efflux in a THP-1 macrophage-derived foam cell line, decreased HDL cholesterol circulation and inhibited reverse cholesterol transport *in vivo* [88]. These events resulted in accelerated pathological progression of atherosclerotic lesions in *apoE^−/−^* mice. The authors concluded *miR-144-3p* as being a key regulator of cholesterol homeostasis, mediating inflammatory reactions in atherogenesis. It is furthermore possible that *miR-144-3p* is a therapeutic target for the treatment of atherosclerosis [88].

### 2.4. Oxidative Stress

Reactive oxygen species (ROS) is a term that includes reactive and partially reduced oxygen metabolites, for example superoxide anion (O_2_−), hydroxyl radicals or hydrogen peroxide (H_2_O_2_) [89]. ROS can cause damage to DNA, proteins and fatty acids, and therefore a redox imbalance resulting from excessive ROS production or insufficient scavenging may lead to cardiovascular disease, for example hypertension, hypercholesterolaemia and atherosclerosis [90,91,92].

Different miRNAs have been identified to play a role in oxidative stress and consecutive endothelial and vascular dysfunction [93]. Several miRNAs are known to be associated with cardiovascular disease, diabetes and obesity due to redox imbalance [93].

For example, up-regulation of *miR-200c*, *miR-141*, *miR-200a*, *miR-200b* and *miR-429*, targeting the zinc finger E-box binding homeobox 1 (*ZEB1*) gene, is associated with apoptosis and senescence caused by H_2_O_2_ in the endothelium [71]. *miR-200c* and *miR-141* up-regulation is associated with obesity, as up-regulation of these miRNAs was identified in the heart of obese rats [94]. Another association with obesity in rats was found for up-regulation of *miR-155*, *miR-183* and *miR-872* [94]. These miRNAs target Heme Oxygenase (Decycling) 1 (*HO-1*), and their up-regulation leads to inflammation, oxidative damage and apoptosis [94]. *miR-200c* and *miR-141* target ribosomal protein S6 kinase, 70 kDa, polypeptide 1 (*S6K1*) which encodes a protein responding to mTOR (mammalian target of rapamycin)-signaling to promote protein synthesis, cell growth, and cell proliferation [95]. *miR-217* was identified to be up-regulated in human atherosclerotic plaques, and targets the *SIRT1* gene, which leads to endothelial dysfunction [96]. Up-regulation of *miR-34*, also targeting *SIRT1*, was found to be associated with myocardial infarction [97].

### 2.5. lncRNAs, Atherosclerosis, Insulin Resistance and Adipogenesis

The chromosome 9p21 locus is the strongest genetic risk factor for atherosclerosis, although this gene locus is not associated with frequent cardiovascular disease risk factors, such as dyslipidaemia and hypertension [98,99,100,101]. Within the risk region, in the locus of the tumor suppressor protein INK4, lies the antisense lncRNA (*ANRIL*) [102,103]. A clear association between *ANRIL* with the chromosome 9p21 genotype has been found in several studies [102,103,104,105,106,107]. It was also demonstrated that *ANRIL* expression positively correlates with atherosclerosis severity [108]. *ANRIL* regulates target-genes, leading to increased cell proliferation, increased cell adhesion and decreased apoptosis, which are known mechanisms of atherogenesis [108]. In a study by Holdt *et al.* [108], *ANRIL-*regulated networks were confirmed in 2280 individuals with and without coronary artery disease, and were functionally validated. It was shown that *ANRIL* isoforms, up-regulated in patients carrying the chromosome 9p21 atherosclerosis risk haplotype, modulate gene networks and lead to pro-atherogenic cellular properties, making them proliferate and forming cell contacts.

Insulin resistance constitutes a key step in the development of metabolic diseases. It was observed that insulin and insulin-like growth factor (IGF) 1 signaling also trigger distinct changes in lncRNA expression, as it was demonstrated for the lncRNA *CRNDE* [109]. Thus, lncRNAs may be involved in the metabolic effects of insulin resistance. The role of the lncRNA *H19* in pancreatic islet development and function was investigated by Ding *et al.*, in 2012 [110]. In this study *H19* was found to be involved in the intergenerational transmission of diabetes mellitus (gestational diabetes mellitus) and in the diabetes-associated impairment of islet structure and function. Intrauterine hyperglycaemia has been suggested to be a determinative factor for diabetes in adulthood [111,112]. Thus, further investigation of the role of ncRNAs in pancreatic islet cell function might contribute to a better understanding of why certain individuals carry a higher risk for diabetes compared to others.

Global lncRNA screening approaches systematically evaluated the lncRNA transcriptome in human pancreatic beta cells, and thereby >1000 lncRNAs were reported [113]. By using RNA sequencing data of 16 non-pancreatic tissues, it was demonstrated that the pattern of pancreatic lncRNAs was significantly more specific for islet cells (40%–55%) than the pattern of protein encoding genes (9.4%) [113]. Up-regulation of islet-specific lncRNAs during glucose stimulation and their dysregulation in patients suffering from type 2 diabetes mellitus pointed to a pathophysiological role of lncRNAs in the integrity of pancreatic tissue [113]. It was found that >1000 lncRNAs of the human beta cell ncRNA transcriptome were expressed in an islet-specific fashion involving islet-specific splicing events and promoter utilization [114]. It remains to be elucidated exactly how lncRNAs mediate beta cell differentiation and function.

Accumulation of excess lipids in white adipose tissue, leading to low-grade inflammation, has been linked to the development of insulin resistance in obese individuals [115,116,117]. lncRNAs are involved in the differentiation of adipose tissue, as for example the lncRNA *SRA*, which is required for full transactivation of the proadipogenic transcription factor Peroxisome proliferator-associated receptor gamma (Pparg) [118]. Furthermore, RNAi-mediated loss-of-function of *SRA* interferes with *in vitro* differentiation of 3T3-L1 preadipocytes [118].

In a study by Sun and colleagues the signatures and specific regulations of 175 lncRNAs during adipogenesis were reported, of which a significant number was enriched in the adipose tissue [119]. In this study, certain lncRNAs that were specifically relevant for adipogenesis were depleted *in vitro* using siRNAs. Distinct lncRNAs were found to be specifically up-regulated during adipogenesis and were induced by the proadipogenic transcription factors *Cebpa* and *Pparg.* These lncRNAs were required for complete maturation of adipocyte progenitor cells. Thus, there is evidence for a crucial role of lncRNAs in the control of adipogenesis and fat cell metabolism [119].

## 3. Discussion

Evidently, a vast variety of non-coding RNAs, mostly miRNAs, play a role in dyslipidaemia and atherogenesis. These non-coding RNAs represent potential therapeutic targets for the treatment of various diseases that are associated with a dysfunctional lipid metabolism. However, the post-transcriptional regulation of genes by these non-coding RNAs is not completely clarified, and the exact mechanisms by which this gene-regulation takes place remain to be elucidated.

Recently it has been revealed that miRNAs are present in the blood plasma, rendering them probably useful as disease biomarkers [27]. Since miRNAs are dysregulated in many diseases, the establishment of circulating miRNAs as biomarkers may be possible for any of these. However, the specificity of a given miRNA as biomarker for a certain disease remains to be investigated, because a miRNA may be involved in many different pathways, and false-positive results have to be avoided.

### miRNAs as Therapeutic Targets

miRNAs can be regulated by administration of miRNA targeting substances, as it has recently been demonstrated in cancer. It was shown in non-human primates that the administration of anti-miRNA oligonucleotides reduces *miR-122* activity in the liver [60]. Suppression of oncogenic *miR-221* by antagonistic miRNAs resulted in prolonged survival and reduction of tumor number and size in a liver-cancer mouse model [120]. Moreover, it was demonstrated that modulating miRNAs can make cancer cells more sensitive to chemotherapeutics [121,122].

*miR-24*, a miRNA that is involved in human carcinogenesis, was demonstrated to be significantly down-regulated in gastric cancer tissue [123]. As retrovirus-mediated gastric cancer cells expressing *miR-24* (SGC-7901/RV-*miR-24*) were injected into nude mice, tumor formation was found to occur much more slowly, compared to controls. Cell proliferation, migration and invasion, cell cycle arrest of tumor cells in G0/G1 phase and increased apoptosis were observed [123]. Thus, *miR-24* functions as a tumor suppressor in gastric cancer cells, and is a potential therapeutic target in the treatment of gastric cancer [123].

The ability to regulate miRNAs opens new options also for the treatment of dyslipidaemia, for example by the delivery of miRNA mimics to enhance miRNA function, or of anti-miRNAs to inhibit miRNA function [124]. The miRNA mimic technology is an approach whereby gene silencing is achieved [125]. Non-natural double-stranded miRNA-like RNA fragments are designed in a way that the 5'-end bears a partially complementary motif to a certain sequence in the 3'UTR (untranslated region) that is unique to the target gene. This RNA fragment is then introduced into cells and mimics an endogenous miRNA. It specifically binds to the target gene, which leads to posttranscriptional repression and inhibition of translation of the selected gene target [125]. The advantage, in comparison to endogenous miRNAs, is the specificity for only one gene [125].

Three different types of chemical modifications have been used to modify miRNA function *in vivo* [126]. Firstly, anti-miRNAs can be linked to cholesterol (antago-miRNA) to facilitate cellular uptake. Secondly, oligonucleotides with locked nucleotides acid (anti-miRNAs) or, thirdly, 2'-*O*-methoxyethyl phosphorothioate modifications may be used [126]. Anti-miRNAs are an approach for miRNA loss-of-function studies, in which chemically modified antisense oligonucleotides are used, which sequester the mature miRNA in competition with cellular target mRNAs. This leads to an inhibition of function of the miRNA, resulting in a de-repression of its targets [127].

*miR-122* was antagonized in mouse liver using these three classes of anti-miRNAs in three independent studies, and revealed *miR-122* antagonism to reduce plasma cholesterol levels in cardiovascular diseases, as for example cardiac hypertrophy, heart failure, arrhythmia and atherosclerosis [45,61,62]. Thus, all of these approaches can be used for therapeutic purposes [126]. For miRNAs that are pathologically down-regulated, miRNA re-expression strategies must be used, whereas anti-miRNA strategies are used to suppress up-regulated miRNAs.

Rayner and colleagues conducted a study where an anti-miRNA oligonucleotide, targeting both *miR-33a* and *miR-33b*, was systemically delivered to African green monkeys. As a result, hepatic ABCA1 expression was increased, and plasma HDL cholesterol levels were sustainably increased over 12 weeks [59]. The expression of several *miR-33b* target genes, all involved in fatty acid oxidation, was also increased, namely *CROT*, *CPT1A*, *HADHB* and *PRKAA1*. The expression levels of genes involved in fatty acid synthesis (*SREBF1*, *FASN*, *ACYL* and *ACACA*) were decreased, resulting in distinct suppression of plasma VLDL-associated TGs [59]. These findings suggest *miR-33a* and *miR-33b* inhibition to be potential therapeutic strategies for the prevention and treatment of atherosclerosis, raising HDL cholesterol and lowering VLDL and TG levels [59].

miRNA inhibitors that can be expressed in cells, so-called miRNA sponges, have been developed as an alternative to chemically modified antisense oligonucleotides [128]. These miRNA sponges are RNAs produced from transgenes, working as competitive miRNA-inhibitors. miRNA sponges are transcripts expressed by strong promoters, which contain multiple binding sites for a given miRNA of interest [128]. Vectors encoding these miRNA sponges can be transfected into cultured cells, and consecutively certain miRNA targets are de-repressed by inhibition of the targeting miRNA [128].

## 4. Conclusions

Vascular diseases and dyslipidaemia are complex and multifactorial diseases, in which many genes are involved.

When considering miRNAs as potential disease biomarkers or therapeutic targets, one must take into account the fact that miRNAs regulate several genes at once. With the manipulation of a given miRNA, the expression and function of many genes may be altered, and thus, harmful and unexpected side effects may occur. When, for therapeutic purposes, a certain gene that is known to be regulated by a certain miRNA, is targeted, it must be kept in mind that a gene is usually regulated not only by one, but by several miRNAs.

Further studies on miRNAs and lncRNAs are necessary to clarify their *in vivo* effect as biomarkers and/or therapeutic targets, and to outline their clinical relevance.
